# Gestational urinary concentrations of glyphosate and aminomethylphosphonic acid in relation to preterm birth: the MIREC study

**DOI:** 10.1038/s41370-024-00702-w

**Published:** 2024-09-18

**Authors:** Jillian Ashley-Martin, Leonora Marro, James Owen, Michael M. Borghese, Tye Arbuckle, Maryse F. Bouchard, Bruce Lanphear, Mark Walker, Warren Foster, Mandy Fisher

**Affiliations:** 1 Environmental Health Research and Science Bureau, Health Canada, Ottawa, ON Canada; 2 Armand-Frappier Santé Biotechnologie Research Centre, Institut National de la Recherche Scientifique, Laval, QC Canada; 3 Faculty of Health Sciences, Simon Fraser University, Burnaby, BC Canada; 4 Department of Obstetrics and Gynecology, University of Ottawa, Ottawa, ON Canada; 5 Department of Obstetrics and Gynecology, McMaster University, Hamilton, ON Canada

**Keywords:** glyphosate, AMPA, preterm birth, pregnancy, biomonitoring, cohort

## Abstract

**Background:**

Few high-quality studies have evaluated associations between urinary glyphosate or its environmental degradate (aminomethylphosphonic acid (AMPA)] and preterm birth (PTB).

**Objectives:**

To quantify associations between urinary glyphosate and AMPA and preterm birth in the pan-Canadian Maternal-Infant Research on Environmental Chemicals (MIREC) study and determine if associations differ by fetal sex.

**Methods:**

We measured first trimester urinary glyphosate and AMPA concentrations in MIREC participants who were recruited between 2008–2011 from 10 Canadian cities. Of the 1880 participants whose first trimester urine samples were analyzed for glyphosate or AMPA, 1765 delivered a singleton, live birth. Our primary outcome was preterm birth (PTB) defined as births occurring between 20 and <37 weeks. Secondary outcomes were spontaneous preterm births (sPTB) and gestational age. We modelled the hazard of PTB and sPTB using discrete time survival analysis with multivariable logistic regression to calculate odds ratios (OR). We used multivariable linear regression models to quantify associations between analytes and gestational age. To assess effect modification by fetal sex, we stratified all models and calculated interaction terms. In the logistic regressions models we additionally calculated the relative excess risk due to interaction.

**Results:**

Six percent (*n* = 106) of the study population delivered preterm, and 4.7% (*n* = 83) had a spontaneous preterm birth. Median specific-gravity standardized concentrations of glyphosate and AMPA were 0.25 and 0.21 µg/L. Associations between both glyphosate or AMPA and PTB, sPTB, and gestational age centered around the null value. The adjusted ORs of PTB for each doubling of glyphosate and AMPA concentrations were 0.98 (95% CI: 0.94, 1.03) and 0.99 (95% CI: 0.92, 1.06) respectively. We observed no evidence of differences by fetal sex.

**Conclusions:**

In this Canadian pregnancy cohort, neither glyphosate nor AMPA urinary concentrations was associated with PTB or reduced gestational length.

## Introduction

Glyphosate, the active ingredient in many herbicide formulations, is the most widely applied pesticide globally [1]. Following the introduction of glyphosate tolerant crops in 1996, the use of glyphosate-based herbicides has increased globally [2] resulting in more opportunities for exposure to glyphosate and its primary environmental degradate - aminomethylphosphonic acid (AMPA) [3]. Routes of non-occupational exposure to glyphosate and AMPA include ingestion of food residues and drinking water, dermal absorption when handling the pesticide or treated surfaces, and inhalation of spray drift [4]. We previously reported that the majority of participants in a Canadian pregnancy cohort had detectable concentrations of glyphosate and AMPA [5]; our findings and those of the Canadian Health Measures Survey [6] suggest that exposure to these chemicals is widespread.

Research into the health effects of glyphosate has largely focused on its potential carcinogenic properties and resulted in divergent conclusions. The World Health Organization International Agency for Research on Cancer declared glyphosate to be ‘probably carcinogenic’ using a hazard-based classification [7, 8]. In contrast, Health Canada and other international pesticide regulatory authorities determined that glyphosate does not pose a carcinogenic risk [9–11]. Glyphosate acts by disrupting an enzyme in the shikimate pathway; this pathway is integral to amino acid production in plants but not humans [1, 12]. Bacteria that reside in the human gastrointestinal tract may, however, contain the shikimate pathway [13–15]. Glyphosate may also be linked to oxidative stress in cell lines via enhanced production of lipid peroxidation and has been shown to induce proinflammatory effects in fish, non-human mammals, and mammals via increased production of pro-inflammatory cytokines and chemokines [16–19].

AMPA may adversely impact plants by producing reactive oxygen species and disrupting chlorophyll biosynthesis [20]. In experimental literature, exposure has been linked to genotoxicity [21] and cytotoxicity in human cells [22]. Authors of epidemiological studies have observed associations between urinary AMPA concentrations and biomarkers of inflammation during pregnancy [23] as well as biomarkers of DNA oxidative damage in children [24].

Enhanced inflammation and oxidative stress during pregnancy may disrupt the tightly regulated balance of immunoregulation necessary to maintain the viability of the developing fetus [25]. Preterm birth is one possible sequelae of dysregulated inflammation and oxidative stress during pregnancy [26–30]. Few high-quality studies with biomonitoring data on glyphosate and AMPA have evaluated associations between these chemicals and either preterm birth (PTB) or reduced gestational age.

The existing epidemiological research on urinary glyphosate and AMPA concentrations and preterm birth is based on few studies of small sample size (*n* = 52–247) all of which took place in the US and territories [31–34]. Authors of the Puerto Rico Test site for Exploring Contamination Threats (PROTECT) study [31], the multi-centre US-based Infant Development and Environment (TIDES) study [32], and a study of Indiana pregnant women [33] all report associations between urinary glyphosate or AMPA concentrations and increased risk of preterm birth, spontaneous preterm birth, or reduced gestational age. In contrast, authors of a pilot study of 26 preterm birth cases and 26 controls residing in South Carolina reported null associations between second trimester urinary glyphosate concentrations and risk of preterm birth [34].

Our objective was to quantify associations between glyphosate and AMPA urinary concentrations and preterm birth in participants of the pan-Canadian MIREC study. By capitalizing on the large sample size in MIREC and broad geographic distribution of our participants, this analysis addresses noted gaps in existing literature. Our secondary objective was to determine if these associations differed by fetal sex. Mothers carrying male fetuses may experience increased risk of PTB and preterm premature rupture of membranes (pPROM) [35, 36]. There may also be sex-specific mechanisms underlying oxidative stress related to PTB [37, 38**]**.

## Methods

### Study population

MIREC is a pregnancy cohort of 2001 participants recruited from 10 Canadian cities (Vancouver, Edmonton, Winnipeg, Sudbury, Ottawa, Kingston, Toronto, Hamilton, Montreal, and Halifax) between 2008 and 2011 [39]. Individuals were eligible for participation if they had no serious medical complications, were at least 18 years old, less than 14 weeks gestation at the time of recruitment and could communicate in either English or French. Participants provided biological specimens as well as information on their sociodemographic characteristics, health history, and lifestyle at clinic visits during each trimester. At the first trimester visit (range: 6–13 weeks), 1880 participants provided a spot urine sample and consented to use of their biological specimens in future research [5]. The present analysis was further restricted to live, singleton births with complete data on gestational age at delivery resulting in a sample size of 1765 (Supplemental Fig. 1).

The MIREC study was reviewed by the Health Canada Research Ethics Board, the research ethics committee at the Study Coordinating Centre (St Justine’s Hospital (Montreal, QC)), and by ethics committees at all recruitment sites. Participants provided informed consent prior to participation.

### Exposure assessment

Urine samples were collected between 6–13 weeks gestation. Urinary glyphosate and AMPA concentrations were quantified by the Centre de Toxicologie du Québec at the Institut National de Santé Publique du Québec (INSPQ) as previously described [5, 40]. Briefly, chemists analyzed urine samples using Ultra-Performance Liquid Chromatography (Waters Acuity) coupled to tandem mass spectrometry (Waters Xevo TQ-XS) (UPLC-MS/MS) in the multiple reaction monitoring mode using an electrospray ionization source in the positive mode. The limits of detection (LOD) and quantification (LOQ) were 0.08 and 0.26 μg/L for glyphosate and 0.09 and 0.29 μg/L for AMPA. The overall quality and accuracy of the analytical method for glyphosate and AMPA were monitored through participation in the following interlaboratory quality assurance programs: German External Quality Assessment Scheme (G-EQUAS; Erlangen, Germany), the External Quality Assessment Scheme for Organic Substances in Urine (OSEQAS; Centre de Toxicologie du Québec (CTQ)/INSPQ, Quebec, Canada) and the Human Biomonitoring for Europe (HBM4EU, Wageningen, Netherlands). Machine readings data were available and used for all measurements below the LOD. In cases where the machine readings values was zero, we used one-half of the next smallest positive value.

### Outcome assessment

Our primary outcome was preterm birth (PTB) defined as a dichotomous measure of births occurring between 20 and <37 weeks. Considering that spontaneous preterm births likely result from inflammation whereas medically indicated preterm birth may result, in part, from aberrant placentation [26], we also conducted analyses examining associations with spontaneous preterm births with the hypothesis that inflammation – rather than aberrant implantation – is a more likely mechanism underlying any potential association between glyphosate and PTB. Consistent with Ferguson et al. [41], we modelled this variable as a dichotomous measure of preterm birth defined as spontaneous labour and/or premature rupture of membranes (pPROM) as noted in their medical chart at delivery. Gestational age at delivery in days was an additional secondary outcome.

### Covariates

We identified potential confounders using previous literature [5, 42]. These variables included maternal age, pre-pregnancy body mass index (BMI), country of birth, race and ethnicity, education, parity, and first trimester smoking status as categorized in Table 1. Covariates were the same for the glyphosate and AMPA models except for race and ethnicity and country of birth. In our previous analysis, we reported that race and ethnicity was associated with urinary AMPA concentrations whereas country of birth was associated with urinary glyphosate concentrations [5]. Participants provided data on all socioeconomic and health history variables at the first trimester clinic visit. Pre-pregnancy BMI was calculated using measured height at the clinic visit and self-reported pre-pregnancy weight. Data on fetal sex, which was evaluated as a potential effect modifier, was abstracted from medical charts at delivery.

**Table 1:** Study population characteristics, singleton livebirths with urinary herbicide and gestational age at delivery data, MIREC, 2008–2001.

		*n*	Median	IQR
Maternal age (years)		1765	32.0	7.0
Gestational age at delivery (weeks)		1765	39.6	1.8
Pre-pregnancy BMI (kg/m^2^)		1636	23.6	5.5

			*n*	Percent
All preterm births			106	6.0
Spontaneous preterm births			83	4.7
Prior preterm birth			116	6.6
Education	High school or less		241	14
	College Diploma		414	24
	University Degree		1108	63
Parity	0		775	44
	1		716	41
	2+		274	16
Smoking status	Never		1082	61
	Former		477	27
	Current/quit when pregnant		205	12
Country of birth	Canada		1428	81
	Other		337	19
Race	White		1468	83
	Non-white		297	17
Infant Sex	Male		934	53
	Female		830	47

*MIREC* Maternal-Infant Research on Environmental Chemicals, *IQR* Interquartile range, Pre-pregnancy BMI *n* = 129, education *n* = 2, smoking status *n* = 1.

### Statistical analysis

We generated descriptive statistics and detection rates for glyphosate and AMPA. To account for urinary dilution, we standardized concentrations by specific gravity (SG) using the following formula [43].

$${P}_{c}={P}_{i}\frac{{{SG}}_{m}-1}{{{SG}}_{i}-1}$$

where *P*_*c*_ is the SG-standardized metabolite concentration, *SG*_*i*_ is the specific gravity of urine sample *i*, and *SG*_*m*_ is the median SG for the cohort. We log_2-_transformed glyphosate and AMPA concentrations prior to regression modelling to reduce the influence of outliers and facilitate calculation of odds ratios (ORs) per doubling in urinary concentrations.

To characterize the hazard probability of PTB and spontaneous PTB between 20 and <37 weeks (36 wks +6 days) of gestation time, we used discrete time survival analysis with logistic regression using 17 indicator variables for each gestational week (20 to 36 completed weeks) at risk of PTB. We fit models in a sequential manner as described by Singer and Willet [44] beginning with Model 1 (Base Model) where PTB is considered to only depend on time (17 gestational weeks at risk). Model 2 builds on the base model by examining the effect of each exposure or covariate in the model, along with an interaction term between covariate or exposure and time variables to determine the validity of the proportionality assumption. We calculated crude and adjusted odds ratios of PTB associated with exposure to glyphosate and AMPA. To assess differences by fetal sex, we stratified analyses by fetal sex, calculated interaction terms, and estimated the relative excess risk due to interaction (RERI) using the method proposed by Knol et al. [45].

To investigate potential associations with gestational age, we assessed the potential for nonlinear trends between each chemical and gestational age using LOESS regression models. Model adequacy was assessed for normality and constant variance of residuals through the following graphical plots: histograms, Q-Q plots for normality and scatter plot of residuals vs predicted values for constant variance. We then used linear regression to model the relationship between log_2_-transformed glyphosate or AMPA and gestational age in days and calculated crude and adjusted beta coefficients.

Complete exposure, outcome and covariate data were available for 87% of the 1765 participants. Exposures or covariates with >1% missing data included pre-pregnancy BMI (7.3%), glyphosate (2.3%), and AMPA (1.6%). We imputed glyphosate or AMPA data for individuals with data for at least one of these analytes (*n* = 1765); individuals missing both glyphosate and AMPA were excluded (*n* = 96). We assumed that missing data were missing at random (MAR) and did not find any major differences across groups of missingness. Multiple Imputation using Chained Equations (MICE) with predictive mean matching (PMM) was used to impute missing exposure and covariates information (m = 50 datasets). PMM involves selecting a data point from the original, non-missing data with a predicted value close to the predicted value of the missing value. All statistical analyses were performed using SAS Enterprise Guide 7.15 (SAS Institute Inc., Cary, NC, 2017).

## Results

Of 1765 participants included in the analysis, 6.0% (*n* = 106) experienced a preterm birth and 4.7% (*n* = 83) experienced a spontaneous preterm birth. Gestational age at delivery ranged from 21.7 weeks to 42.4 weeks with a median of 39.6 weeks (IQR: 38.6, 40.4 weeks). Median maternal age was 32 years. Most participants had university degrees (63%), never smoked (61%), were born in Canada (81%) and self-reported their race and ethnicity as White (83%) (Table 1). Geometric means of SG standardized glyphosate and AMPA concentrations in urine were 0.11 µg/L and 0.16 µg/L, both of which were below the analyte LOQ (Supplementary Table 1). Geometric means of SG standardized urinary concentrations of glyphosate and AMPA were similar between subjects with preterm and term births (glyphosate PTB: 0.10 µg/L (95% CI: 0.05, 0.17) vs term: 0.11 µg/L (95% CI: 0.10, 0.13); AMPA PTB: 0.16 µg/L (95% CI: 0.11, 0.22) vs term: 0.16 µg/L (95% CI: 0.15, 0.17)).

Crude and adjusted ORs of both PTB and spontaneous PTB associated with urinary concentrations of glyphosate and AMPA were centered around the null value (Table 2). We observed no differences in ORs when the models were stratified by fetal sex (Supplementary Table 2) and there was no evidence of interaction based on the RERIs (Supplementary Table 3) or the interaction term. Upon inspection of LOESS curves, we determined that the linearity assumption was met. Crude and adjusted coefficients for log_2_ transformed, SG-standardized urinary concentrations of glyphosate and AMPA with respect to gestational age were close to the null value (Table 3). We observed no evidence of interaction between fetal sex and either urinary concentrations of glyphosate or AMPA (glyphosate β for interaction = 0.02, 95% CI: -0.28, 0.31; AMPA β for interaction = 0.26, 95% CI: −0.20, 0.71), and no differences in results stratified by fetal sex (Supplementary table 4).

**Table 2:** Association between first trimester urinary concentrations of glyphosate and AMPA (µg/L) and odds of preterm birth.

	All preterm birth (*n* = 106)				Spontaneous preterm birth (*n* = 83)			
	Crude OR	95% CI	Adjusted OR^a^	95% CI	Crude OR	95% CI	Adjusted OR^b^	95% CI
Glyphosate	0.99	0.94, 1.03	0.98	0.94, 1.03	0.98	0.93, 1.03	0.98	0.93, 1.03
AMPA	0.99	0.92, 1.07	0.99	0.92, 1.06	1.00	0.91, 1.08	0.99	0.91, 1.08

^a^adjusted for maternal age, pre-pregnancy BMI, education, parity, smoking and country of birth.

^b^adjusted for maternal age, pre-pregnancy BMI, education, parity, smoking and race/ethnicity.

Glyphosate and aminomethylphosphonic acid (AMPA) are both specific gravity-standardized and log_2_ transformed.

**Table 3:** Linear regression models of each doubling of first trimester urinary concentrations of glyphosate and AMPA (µg/L) and gestational age at birth in days (n = 1765).

	Crude β	95% CI	Adjusted β	95% CI
Glyphosate^a^	0.00	−0.15, 0.15	0.00	−0.14, 0.15
AMPA^b^	0.01	−0.21, 0.24	0.06	−0.17, 0.28

^a^adjusted for maternal age, pre-pregnancy BMI, education, parity, smoking and country of birth.

^b^adjusted for maternal age, pre-pregnancy BMI, education, parity, smoking and race/ethnicity.

Glyphosate and aminomethylphosphonic acid (AMPA) are both specific gravity-standardized and log_2_ transformed.

When a complete case analysis was performed without multiple imputation, results did not differ for either the logistic or linear regression models.

## Discussion

In this cohort of Canadian pregnant people, we observed no associations between first trimester urinary concentrations of glyphosate or AMPA and preterm birth or gestational age. Results did not differ when we restricted to spontaneous preterm births or stratified by fetal sex.

We identified one other epidemiological study reporting a null association between glyphosate and PTB, a pilot case-control study of 26 cases and 26 controls with median glyphosate concentrations below the LOD used in the MIREC study [34] (Supplementary Table 5). Our null findings differ from positive associations reported by authors of the TIDES [32] and PROTECT studies [31]. These discrepancies could be explained – in part – by lower glyphosate concentrations in MIREC than PROTECT and the earlier timing of urine collection in MIREC than TIDES and PROTECT. Median SG- standardized glyphosate concentrations in MIREC (0.25 µg/L) were comparable with those observed in the TIDES study (0.25 µg/L) but half of that reported in the PROTECT study (visit 1: 0.50, visit 3: 0.47 µg/L). Median MIREC specific gravity standardized AMPA concentrations (0.21 µg/L) were only slightly lower than observed in the PROTECT study (0.26, 0.23 µg/L) and higher than observed in TIDES (0.16 µg/L) suggesting that factors other than differential exposure levels are contributing to the differences in associations.

Timing of urine collection is one possible factor. Both the TIDES [32] and PROTECT [31] study collected urine samples in the second trimester whereas the MIREC samples were collected in early pregnancy (6 – 12 weeks). Associations between urinary glyphosate and AMPA concentrations and PTB in PROTECT were only evident with visit 3 (mean gestational age = 26 weeks) samples; associations between the visit 1 concentrations (mean gestational age=18 weeks) and PTB were null. It is possible that the first trimester glyphosate and AMPA concentrations in our study do not reflect exposure during a potential critical window physiologically relevant to mechanisms underlying preterm birth, such as inflammation or oxidative stress. The short half-lives ( <24 h) of glyphosate and AMPA [3, 46] and low correlation between visit 1 and visit 3 concentrations in PROTECT (glyphosate ρ = 0.36, AMPA ρ = 0.19) [31] suggest that first trimester concentrations cannot be used as a proxy for exposure throughout pregnancy.

A growing body of experimental [17, 47, 48] and epidemiological studies support the hypothesis that glyphosate and AMPA exposure may be linked to inflammation and oxidative stress. First, in these studies, glyphosate and AMPA appear to be more strongly associated with spontaneous PTB – which is more likely to be driven by inflammation and oxidative stress – than medically indicated PTB. For example, in the PROTECT study, the magnitude of effect increased from 67% to 88% higher odds with each IQR increase in AMPA when the study population was restricted to spontaneous births. Similar observations were seen with glyphosate but with a smaller magnitude of effect [31]. In the TIDES study, Lesseur et al. observed positive associations between glyphosate and AMPA and spontaneous birth modelled as a continuous measure of gestational age but null associations with dichotomous PTB. In their study, results were similar between glyphosate and AMPA [32].

The potential association between glyphosate and AMPA and oxidative stress is further elucidated in a separate analysis of the PROTECT study by Eaton et al. [23]. These authors reported that an interquartile increase in visit 3 AMPA concentrations was associated with 9.5% higher levels of a metabolite of 8-isoprostane-prostaglandin-F2α. Associations between glyphosate and the biomarkers were of similar direction but lower magnitude. Associations between visit 1 chemical concentrations and oxidative stress biomarkers were close to the null providing further evidence to suggest that late second trimester exposures may be associated with oxidative stress rather than first or early second trimester exposures. Oxidative stress may be a mechanism underlying subsequent pregnancy complications such as preterm birth [26–30]. Eaton et al. [23] note that further study is necessary to better characterize the timing of glyphosate and AMPA exposures in relation to prenatal oxidative stress and subsequent pregnancy complications. In an analysis of 177 children age 10-11 years in the Cyprus ORGANIKO study, Makris reported positive associations between AMPA – but not glyphosate - and concurrently measured 8-hydroxy-2′-deoxyguanosine (8-OHdG), a biomarker of oxidative DNA damage [24] providing evidence that AMPA may be linked to oxidative stress in non-pregnant populations.

It is also possible that differences between our findings and those reported by PROTECT [31] and TIDES [32] are due to the socioeconomic profile of participants or differences in covariates in adjusted models. PROTECT study participants reside in a region of known contamination due to the presence of Superfund waste sites on the island, are more likely to smoke and have lower household incomes than MIREC participants. In contrast, MIREC participants generally reside in Canadian cities, and are non-smokers, of moderate to high socioeconomic status. The TIDES study participant demographics are more similar to MIREC; however, it is possible that the findings reported by Lesseur et al. are subject to some degree of confounding bias as their models were not adjusted for either smoking or pre-pregnancy BMI [32]. Due to the case-control study design of the PROTECT and TIDES studies, we cannot compare rates of preterm birth to that observed in MIREC. The percent of preterm births in MIREC (6%) is, however, lower than observed in Canada (8.0-8.2%) during the years of MIREC recruitment [49]. This lower than average rate of PTB in MIREC may have lessened our ability to identify an association.

It is difficult to compare our results to the two other studies that reported positive associations due to differences in study design [33, 50]. Parvez et al. reported an inverse correlation between glyphosate and lower gestational age in 71 pregnant people from Indiana. The urinary glyphosate concentrations were 13 times higher than in MIREC and samples were collected throughout pregnancy [33]. Ling et al. observed modest (4–%) increases in odds of PTB for ever vs never exposure to glyphosate estimated via ambient air exposure data from the first and second trimester [50] but this study did not include any biomonitoring data. In contrast, our results are consistent with the pilot nested case-control study by Varde et al. [34] but their results may have been subject to type 2 error due to the small sample size (*n* = 26 cases, 26 controls).

Our analysis benefited from the large sample size of the MIREC and availability of biomonitoring data in approximately 94% of the original cohort. This large sample size enabled us to examine potential differences in associations by fetal sex. Ling et al. in their analysis of estimated ambient glyphosate exposure and PTB observed modestly higher OR of PTB among pregnancies carrying female fetuses compared to male fetuses [50]. Their findings may not reflect true underlying sex-specific differences as all confidence intervals were overlapping and the authors did not report formal tests for interaction. No other epidemiological study that estimated exposure using urinary concentrations of glyphosate or AMPA investigated sex-specific differences. Last, as a result of the minimal sociodemographic heterogeneity in MIREC confounding bias due to socioeconomic status is minimal; the availability of sociodemographic data allowed us to further minimize this potential bias through statistical adjustment.

Our findings are limited by reliance on one first trimester spot urine sample. As previously noted, first trimester concentrations are not reflective of exposure throughout pregnancy and may not reflect the most relevant physiological window. Serial measurements with a standardized urine collection time would enhance the rigor of our exposure assessment. Nevertheless, considering the importance of early gestation in the development of key vital organs [51], our results – if confirmed in future studies – are a valuable contribution towards understanding potential associations between early trimester glyphosate and AMPA exposure and pregnancy complications. Restricting the study population to live, singleton births has the potential to introduce live birth bias; if either glyphosate or AMPA urinary concentrations were associated with fetal loss, the most vulnerable fetuses would be excluded from our study sample due to a competing risk. Due to the small number of fetal losses in MIREC (spontaneous abortions and stillbirths *n* = 33), live birth bias is likely negligible in our analysis. Our results are also limited by the focus on glyphosate as the primary exposure. Chemical herbicide formulations contain preservatives and adjuvants and, by focusing on glyphosate only, we were not able to capture any potential additive or synergistic effects that may results from exposure to the herbicide formulation as a whole [22]. Last, although the minimal sociodemographic heterogeneity minimizes confounding, it also minimizes generalizability. As noted, our findings differed from those reported in the PROTECT study underscoring the value of conducting research in multiple populations and investigating mechanisms underlying the observed associations.

## Conclusions

In this population of Canadian pregnancy cohort, we observed no associations between either glyphosate or AMPA urine concentrations and risk of preterm birth. These null associations persisted when we restricted the study population to spontaneous pre-term births, examined gestational age as a continuous outcome, and stratified by fetal sex. Our reported urinary concentrations are relevant to the time of MIREC recruitment (2008–2011) in a largely urban pregnant population of moderate to high socioeconomic status. Continued research examining contemporary exposure levels and in populations of differing sociodemographic profiles is needed to confirm our observed absence of associations.

## Supplementary information

Supplementary Information

## Data Availability

The MIREC Biobank was the sole source of data and biological specimens used in this research. Researchers interested in accessing these data may apply to the MIREC Biobank: https://www.mirec-canada.ca/en/research/.
