# Supplementary material for: Gestational urinary concentrations of glyphosate and aminomethylphosphonic acid in relation to preterm birth: the MIREC study
Source: J Expo Sci Environ Epidemiol. 2024 Sep 18;35(5):876–81. doi: 10.1038/s41370-024-00702-w (PMC12401726; doi:10.1038/s41370-024-00702-w)
Supplement: Supplementary file 1 — Supplementary Information [file 41370_2024_702_MOESM1_ESM.docx]

Supplemental table 1. Descriptive statistics of herbicides^1^ (µg/L) in first trimester urine samples, MIREC study, 2008 – 2011.

|  | |  | n | LOD | <LOD^2^ | LOQ | 25th %ile | Median^3^ | 75th %ile | 95th %ile | Max | Geometric Mean^3^ (95% CI) | |
| --- | --- | --- | --- | --- | --- | --- | --- | --- | --- | --- | --- | --- | --- |
| Not SG-standardized | | | |  |  |  |  |  |  |  |  |  | |
|  | | Glyphosate | 1719 | 0.08 | 26% | 0.26 | <LOD | 0.223 | 0.516 | 1.681 | 7.813 | 0.096 (0.084, 0.111) | |
|  | | AMPA | 1736 | 0.09 | 28% | 0.29 | <LOD | 0.195 | 0.463 | 1.249 | 5.725 | 0.138 (0.126, 0.152) | |
| SG-standardized | | | |  |  |  |  |  |  |  |  |  | |
|  | | Glyphosate | 1716 |  |  |  | 0.117 | 0.247 | 0.461 | 1.091 | 3.907 | 0.111 (0.097, 0.126) | |
|  | | AMPA | 1733 |  |  |  | 0.111 | 0.212 | 0.384 | 0.870 | 6.139 | 0.158 (0.146, 0.172) | |
|  | ^1^ Machine readings were used for results below the limit of detection; values below the limit of detection should be interpreted with caution.  ^2^Detection rates are calculated from the raw data so are not reported for the SG-standardized data.  ^3^Please note that median and geometric mean concentrations are below the LOQ. | | | | | | | | | | | |  |
|  | SG: specific gravity; LOD: limit of detection; AMPA: aminomethylphosphonic acid | | | | | | | | | | | |  |

Supplemental table 2. Association between each doubling of first trimester urinary concentrations of glyphosate and AMPA (µg/L) and odds of preterm birth stratified by fetal sex

Males

|  | All preterm birth | | | | | Spontaneous preterm birth | | | | |
| --- | --- | --- | --- | --- | --- | --- | --- | --- | --- | --- |
|  | n | Crude OR | 95% CI | Adjusted OR | 95% CI | n | Crude OR | 95% CI | Adjusted OR | 95% CI |
| Glyphosate^1^ | 58 | 0.99 | 0.93,1.06 | 0.99 | 0.93, 1.06 | 48 | 0.98 | 0.92,1.05 | 0.98 | 0.92, 1.05 |
| AMPA^2^ | 58 | 0.97 | 0.88,1.07 | 0.97 | 0.88, 1.07 | 48 | 0.99 | 0.88, 1.10 | 0.99 | 0.88, 1.11 |

Females

|  | All preterm birth | | | | | Spontaneous preterm birth | | | | |
| --- | --- | --- | --- | --- | --- | --- | --- | --- | --- | --- |
|  | n | Crude OR | 95% CI | Adjusted OR | 95% CI | n | Crude OR | 95% CI | Adjusted OR | 95% CI |
| Glyphosate^1^ | 48 | 0.97 | 0.91,1.04 | 0.98 | 0.91, 1.04 | 35 | 0.98 | 0.9,1.06 | 0.99 | 0.91, 1.07 |
| AMPA^2^ | 48 | 1.02 | 0.91, 1.15 | 1.01 | 0.90, 1.13 | 35 | 1.0 | 0.88,1.15 | 1.0 | 0.88, 1.14 |

^1^ adjusted for maternal age, pre-pregnancy BMI, education, parity, smoking and country of birth

^2^ adjusted for maternal age, pre-pregnancy BMI, education, parity, smoking and race/ethnicity

Glyphosate and aminomethylphosphonic acid (AMPA) are both specific gravity-standardized and log_2_ transformed

Supplemental table 3. Adjusted interaction terms and relative excess risk due to interaction (RERI) for odds of PTB and spontaneous PTB associated with glyphosate and AMPA (µg/L), by fetal sex.

|  | All preterm births | | Spontaneous preterm births | |
| --- | --- | --- | --- | --- |
|  | β^3^ (95% CI) | RERI (95% CI) | β^3^ (95% CI) | RERI (95% CI) |
| Glyphosate^1^ | 0.46 (-0.54, 1.46) | 0.43 (-0.54, 1.15) | 0.64 (-0.46, 1.73) | 0.53 (-0.54, 1.28) |
| AMPA^2^ | -0.21 (-1.47, 1.04) | -0.20 (-1.23, 0.70) | -0.16 (-1.73, 1.41) | -0.08 (-1.19, 0.70) |

Calculate RERI based on adjusted models:

^1^ adjusted for maternal age, pre-pregnancy BMI, education, parity, smoking and country of birth

^2^ adjusted for maternal age, pre-pregnancy BMI, education, parity, smoking and race/ethnicity

^3^ β coefficient of the interaction term between glyphosate/aminomethylphosphonic acid (AMPA) and fetal sex. Glyphosate and AMPA are both specific gravity -standardized

Supplemental table 4. Association between each doubling of first trimester urinary concentrations of glyphosate and AMPA (µg/L) and gestational age at birth in days by fetal sex

Males

| Analyte | Gestational age | | | | |
| --- | --- | --- | --- | --- | --- |
|  | n | Crude β | 95% CI | Adjusted β | 95% CI |
| Glyphosate^1^ | 935 | 0.02 | -0.19, 0.22 | 0.01 | -0.20, 0.22 |
| AMPA^2^ | 935 | 0.14 | -0.19, 0.47 | 0.18 | -0.15, 0.51 |

Females

| Analyte | Gestational age | | | | |
| --- | --- | --- | --- | --- | --- |
|  | n | Crude β | 95% CI | Adjusted β | 95% CI |
| Glyphosate^1^ | 830 | -0.02 | -0.22, 0.19 | 0.00 | (-0.21, 0.20) |
| AMPA^2^ | 830 | -0.11 | -0.42, 0.2 | -0.08 | (-0.38, 0.23) |

^1^ adjusted for maternal age, pre-pregnancy BMI, education, parity, smoking and country of birth

^2^ adjusted for maternal age, pre-pregnancy BMI, education, parity, smoking and race/ethnicity

Glyphosate and aminomethylphosphonic acid (AMPA) are both Specific gravity-standardized and log2 transformed

| Supplemental table 5. Cohort studies examining glyphosate, AMPA and preterm birth | | | | | | |
| --- | --- | --- | --- | --- | --- | --- |
| Study  (Years of Recruitment) | Sample Size | Timing of urine collection | Median glyphosate concentrations (µg/L) | Median AMPA concentrations (µg/L) | Main findings |  |
| MIREC^1^  10 cities, Canada  (2008-2011) | 1765 | 6-13 weeks | 0.25^1^ | 0.21^1^ | Null associations between glyphosate, AMPA and PTB, sPTB, and GA |  |
| Lesseur et al, 2022^2^  TIDES, 4 cities, USA  (2010-2012) | 69 PTB  94 term | 20.6 ±3.2 weeks | 0.25 | 0.16 | HR of spontaneous PTB:  Glyphosate: 1.31 (95% CI: 1.00-1.71)  AMPA: 1.32 (95% CI: 1.00-1.73)  OR of PTB:  Glyphosate: 1.55 (95% CI: 0.97, 2.57)  AMPA: 1.06 (95% CI: 0.71, 1.58) |  |
| Silver al, 2022^3^  PROTECT, Northern Karst aquifer region, Puerto Rico  (2011-2017) | 53 cases  194 controls | Visit 1:18±2  Visit 3: 26±2  weeks | Visit 1: 0.50  Visit 3: 0.47 | Visit 1: 0.26  Visit 3: 0.23 | Visit 1 OR of PTB  Glyphosate: 1.11 (95% CI: 0.71, 1.74)  AMPA: 0.92 (95% CI : 0.67,1.27)  Visit 3 OR of PTB  Glyphosate: 1.35 (95% CI: 0.99, 1.83)  AMPA: 1.67 (95% CI: 1.26, 2.20) |  |
| Varde et al, 2023^4^  1 city, South Carolina, USA  (2011-2014) | 26 cases  26 controls | 18-22 weeks | Cases 0.06  Controls: 0.05 |  | OR and PTB  Glyphosate: 1.06 (95% CI: 0.61, 1.86) |  |
| Parvez et al, 2018^5^  Central Indiana, USA  (2015-2016) | 71 | 11-38 weeks | 3.25 |  | Correlation between glyphosate and gestational age: r = - 0.28, p = 0.02 |  |

Median concentrations are specific gravity standardized

1. Median glyphosate and AMPA concentrations are below the LOQ.

1. Ashley-Martin, J. *et al.* Urinary concentrations and determinants of glyphosate and glufosinate in pregnant Canadian participants in the MIREC study. *Environ. Res.* **217**, 114842 (2023).

2. Lesseur, C. *et al.* Maternal urinary levels of glyphosate during pregnancy and anogenital distance in newborns in a US multicenter pregnancy cohort. *Environ. Pollut.* **280**, 117002 (2021).

3. Silver, M. *et al.* Prenatal exposure to glyphosate and its environmental degradate, aminomethylphosphonic acid (Ampa), and preterm birth: A nested case-control study in the protect cohort (puerto rico). *Environ. Health Perspect.* **129**, 1–11 (2021).

4. Varde, M. *et al.* Glyphosate exposure and preterm birth: A nested case-control pilot study. *Reprod. Toxicol.* **117**, 108350 (2023).

5. Parvez, S. *et al.* Glyphosate exposure in pregnancy and shortened gestational length: A prospective Indiana birth cohort study. *Environ. Heal. A Glob. Access Sci. Source* **17**, 1–12 (2018).

Supplemental Figure 1. Study Flow Diagram

2001 participants recruited

18 withdrew

43 did not consent to biobank

60 did not provide first trimester urine samples

1880 biobanked first trimester urine samples analyzed for glyphosate and AMPA

115 removed due to multiple birth, stillbirth, spontaneous abortion, therapeutic abortion, or lost to follow-up

1719 glyphosate data

1736 AMPA data

1765 live, singleton births with either glyphosate or AMPA data

106 preterm births

83 spontaneous preterm births
